# SuRxgWell: study protocol for a randomized controlled trial of telemedicine-based digital cognitive behavioral intervention for high anxiety and depression among patients undergoing elective hip and knee arthroplasty surgery

**DOI:** 10.1186/s13063-023-07634-0

**Published:** 2023-11-09

**Authors:** A. Murat Kaynar, Charles Lin, Andrea Gomez Sanchez, Danielle R. Lavage, Amy Monroe, Nicole Zharichenko, Meredith Strassburger, Katheryn Saucier, Yram J. Groff, Brian A. Klatt, Michael J. O’Malley, Eva Szigethy, Ajay D. Wasan, Jacques E. Chelly

**Affiliations:** 1 Department of Anesthesiology and Perioperative Medicine, University of Pittsburgh, Pittsburgh, PA USA; 2 The Center for Innovation in Pain Care (CIPC), University of Pittsburgh, Pittsburgh, PA USA; 3 Department of Critical Care Medicine, University of Pittsburgh, Pittsburgh, PA USA; 4 The Clinical Research, Investigation, and Systems Modeling of Acute Illness (CRISMA) Center, University of Pittsburgh, Pittsburgh, PA USA; 5 Department of Psychiatry, University of Pittsburgh, Pittsburgh, PA USA; 6 Department of Orthopaedic Surgery, University of Pittsburgh, Pittsburgh, PA USA

## Abstract

**Background:**

Mood disorders (anxiety, depression), sleep disorders, and catastrophizing lead to increased post-operative pain perception, increase in postoperative opioid consumption, decreased engagement with physical activity, and increased resource utilization in surgical patients. Psychosocial disorders significantly affect postoperative outcome. Unfortunately, studies focused on perioperative psychological assessment and treatment are scarce. We propose to test whether digital cognitive behavioral intervention (dCBI) can help surgical patients. dCBI such as RxWell™ is a proven treatment for mood disorders in medical patients such as reducing depression in patients with inflammatory bowel disease. We hypothesize that RxWell™ will also be effective in surgical patients. This study aims to test whether RxWell™ can improve preoperative mood disorders and subsequently reduce postoperative pain and opioid requirement in patients scheduled for primary total hip and knee arthroplasty (THA, TKA). We named the trial as the SuRxgWell trial.

**Methods:**

This is a randomized, controlled trial that will enroll primary and unilateral THA or TKA patients with anxiety and/or depression symptoms before surgery to receive the SuRxgWell dCBI program and investigate its impact on postoperative outcomes including postoperative pain, anxiety, depression, sleep disorder, and catastrophizing. After signing an informed consent, subjects will be screened using the PROMIS questionnaires, and subjects with a T-score of ≥ 60 on the short Patient-Reported Outcomes Measurement Information System (PROMIS) 4a Anxiety and/or short PROMIS 4a Depression questionnaires will be randomized to either usual care (control group) or the cognitive behavioral intervention, *RxWell*™, plus usual care (intervention group). The control group will receive information on how to locate tools to address anxiety and depression, whereas the intervention group will have access to SuRxgWell 1 month prior to surgery and up to 3 months after surgery. The allocation will be 3:1 (intervention to control). Investigators will be blinded, but research coordinators approaching patients and research subjects will not.

The primary outcome will be day of surgery anxiety or depression symptoms measured with the PROMIS Short Form v1.0 -Anxiety 4a/Depression and Generalized Anxiety Disorder Measure (GAD-7) and Patient Health Questionnaire (PHQ-8). Secondary end points include measuring other health-related quality of life outcomes including sleep disturbance, fatigue, ability to participate in social roles, pain interference, cognitive function, pain catastrophizing, and physical function. Other secondary outcomes include collecting data about preoperative and postoperative pain scores, and pain medication usage, and orthopedic functional recovery at baseline, day of surgery, and 1, 2, and 3 months after the surgery with the Pain Catastrophizing Scale, the Knee injury and Osteoarthritis Outcome Score (KOOS), and Hip injury and Osteoarthritis Outcome Score (HOOS). In addition, subjects will be asked to complete a GAD-7 and PHQ-8 questionnaires bi-weekly (via the *RxWell*™ app for the interventional group or REDCAP for the control group). Data about postsurgical complications, and resource utilization will also be recorded. We will also receive monthly reports measuring the usage and engagement of RxWell use for each participant randomized to that arm. The primary hypotheses will be assessed with intention-to-treat estimates, and differences in primary outcome will be tested using independent two sample *t*-tests. This trial is registered to the ClinicalTrials.gov database (NCT05658796) and supported by the DAPM, UPMC Health Plan, and the NIH.

**Discussion:**

Our trial will evaluate the feasibility of digital cognitive behavioral intervention as a perioperative tool to improve anxiety and depression before and after major orthopedic surgery in comparison to education. If digital cognitive behavioral intervention proves to be effective, this might have important clinical implications, reducing the incidence of chronic postsurgical pain and improving outcomes.

**Supplementary Information:**

The online version contains supplementary material available at 10.1186/s13063-023-07634-0.

## Background

The optimization of perioperative care reduces postoperative complications and undesirable sequelae of surgery such as pain, fatigue, depression, resource utilization, and prolonged convalescence [1]. Patients scheduled for surgery often present with co-occurring mood disorders, such as generalized anxiety, major depression, and/or high levels of pain catastrophizing [2]. These are particularly salient among patients scheduled for primary total hip and knee arthroplasty (THA, TKA), procedures that are expected to increase by 71%, to 635,000 procedures, by 2030 (THA) and by 85%, to 1.26 million procedures, by 2030 (TKA) [3].

Almost 30% of patients undergoing a TKA surgery experience psychological distresses preoperatively [4]. These emotional conditions negatively impact postoperative pain and opioid consumption, post-surgical complications rate, recovery time, and re-hospitalization rate [5–14]. Although preliminary data demonstrate that advances in perioperative medicine such as the use of multimodal and multidisciplinary interventions appear to control perioperative surgical stress response and improve postoperative outcomes, this is insufficient. Optimization of physiology, such glycemic control in diabetic patients or blood pressure management in patients with hypertension, is systematically performed, and yet surgical outcomes can be improved. An often-overlooked opportunity for improving surgical care is optimizing a patient’s preoperative emotional condition. The pathophysiological link between psychological factors and surgical outcomes has been widely described [15]. Unfortunately, studies focusing on perioperative psychological assessment and treatment are lacking. Non-physical preoperative patient factors may directly influence the neuroendocrine and inflammatory response to surgical stress, impacting on immune function and healing, the development of cardiovascular diseases, and neurological events [16–19].

Surgical patients with mood disorders result in longer in-hospital length of stays, higher incidence and odds of readmission, higher rate of medical and implant-related complications, and higher day of surgery and 90-day peri-surgical costs [20, 21]. Mood disorders also increase peri-operative pain, opioid requirement, and delay recovery. The high prevalence of anxiety and depression in patients undergoing total hip and knee makes mood disorders meaningful potential targets for preoperative patient optimization [22]. However, studies analyzing the impact of a preoperative psychological assessment and intervention in orthopedic surgery are missing. Mood disorders can be efficiently assessed using brief screening methods such as the Hospital Anxiety and Depression Scoring (HADS) or PROMIS (Patient-Reported Outcomes Measurement Information System). The PROMIS library has short forms for depression and anxiety and has the added advantage of being validated across different patient populations. Interventions focusing on managing mood disorders could be implemented to improve perioperative outcomes. The FDA recommends cognitive-behavioral intervention (CBI) as a first-line treatment for the non-pharmacologic treatment of depression and anxiety, and this could be a potential intervention for reducing mood disorders and pain levels in surgical patients [23–25]. In-person CBI has been described as an effective tool for reducing the Pain Catastrophizing Scale score and Postoperative General Anxiety Disorder-7 scores; decreasing postoperative pain, opioid use, and length-of-stay at the hospital; and increasing functionality based on Knee Outcome Survey – Activities of Daily Living scale (KOS-ADL) in patients undergoing TKA [26–28]. However, multiple barriers exist preventing surgical patients from accessing CBI, such as high costs and lack of accessibility in remote areas [29].

Telemedicine represents increasingly common strategies for patient care. Similarly, digital Cognitive Behavioral Intervention (dCBI) could be an effective strategy for overcoming some of these barriers. There is preliminary data suggesting that dCBI could be an effective treatment for depression and anxiety in primary care [30]. However, the implementation of dCBI for reducing perioperative psychological and psychosomatic conditions is limited. RxWell™ is an app that includes evidence-based cognitive behavioral techniques and can be used remotely to teach coping strategies based on CBI. It has proven to successfully help patients with inflammatory bowel disease [31].

We are using the RxWell™ dCBI in this clinical trial among orthopedic patients and named the trial as SuRxgWell. In this SuRxgWell trial, we hypothesize that the use of RxWell™ will reduce the deleterious impact of established mood disorders on recovery following primary total hip and total knee replacements by improving the following postoperative outcomes:

- Pain and opioid requirement,
- Improved functional recovery,
- Decrease complications, and
- Reduce resource utilization such as hospital length of stay, postoperative need for visits, and the use of rehabilitation.

## Materials and methods

### Study design

**Fig. 1:**
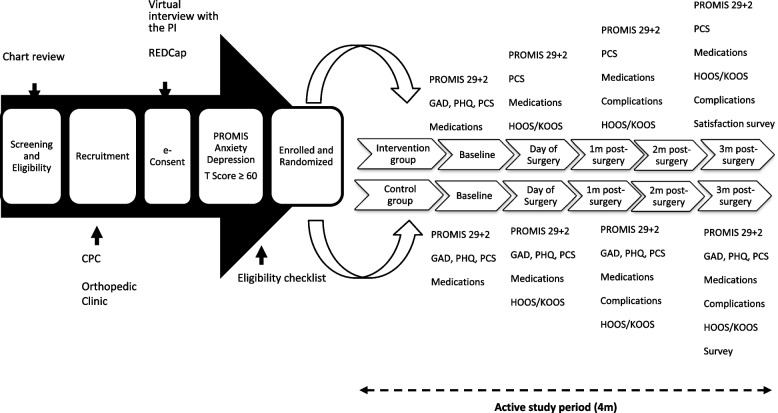
Study workflow

This is a prospective, randomized, controlled clinical trial in a multi-hospital health care system among patients with high levels of anxiety with an indication for primary total hip or knee arthroplasty. We will be comparing two groups: one that receives access to RxWell™ and usual care and one that only receives the usual care. Figure 1 and Table 1 show the study design, timing of pre- and postoperative use of RxWell™, and follow-up evaluations. CONSORT 2010 items and CONSORT-SPI 2018 checklist are presented for the abstract (Table 2) and main text (Table 3). The SPIRIT Checklist is presented as an additional file (Additional file 1). We will recruit patients from 4 hospitals within the *UPMC system* (*UPMC Shadyside*, *Magee*, *East*, and *Passavant hospitals*). In this pilot, randomized feasibility trial, we will prospectively allocate 34 subjects in the control group, and using a 1:3 allocation, we will have 102 in the intervention group (two-sided *t*-test with alpha of .05, power of .8, with a 3:1 ratio and a 50% drop out rate for a Cohen’s *d* of .8). A block randomization schedule generated using the R software (version 4.2.1, R Core Team, 2022) will be used to allocate the participants to either dCBI plus usual care (*intervention*) or usual care (*control*) with a 3:1 allocation (Table 2). Investigators will be blinded for group assignment in REDCap, and they will also be blinded for the monthly reports. Research coordinators, the statistical data analysts, and subjects will not be blinded. To make sure investigators are blinded to the intervention, investigators will not have access to the randomization table and surveys in REDCap. Investigators will only be able to access the eligibility checklist and informed consent surveys in REDCap. Patients will be randomized immediately after the PROMIS Anxiety 4a and the PROMIS Depression 4a patient assessment by a research coordinator using REDCap. We do not anticipate any requirement for unblinding, but if required, the director of clinical research operations, regulatory specialist, study coordinators, or the principal investigator will have access to group allocations and any unblinding will be reported.

**Table 1:** Study design, timing of pre- and postoperative use of RxWell™, and follow-up evaluations

Outcomes	Instrument	Study period	*Eligibility assessment*	*Enrollment*	*Surgery*	*Post-Surgery*		
		*Time*	*After consenting the patient*	*Baseline (3–4 weeks before surgery)*		*1M*	*2M*	*3M*
*Primary outcomes*								
Self-reported anxiety	*PROMIS 4a anxiety*		x	x	x	X	x	x
	*GAD-7*			x^a^	x^a^	x^a^	x^a^	x^a^
Self-reported depression	*PROMIS 4a depression*		x	x	x	x	x	x
	*PHQ-8*			x^a^	x^a^	x^a^	x^a^	x^a^
*Secondary outcomes*								
Sleep disturbances, fatigue, ability to participate in social roles	*PROMIS 29+2 (Except PROMIS 4a Anxiety and 4a Depression)*			x	x	x	x	x
Pain catastrophizing	*Pain Catastrophizing Scale*			x	x	x	x	x
Perioperative pain	*Pain Scores*			x	x	x	x	x
Opioid and non-opioid consumption				x	x	x	x	x
Functionality	*HOOS/KOOS*			x	x	x	x	x
Post-surgical complications						x	x	x
Patient satisfaction								x

^a^The intervention group will also be assessed bi-weekly with GAD-7 and PHQ-8 through the RxWell app

**Table 2:** CONSORT 2010 items and CONSORT-SPI 2018 checklist for the abstract

Section	CONSORT abstract item	Relevant CONSORT-SPI item	Reported on page #
Title	Identification of the study as randomized		1
Authors	Contact details for the corresponding author		1–2
Trial design	Description of the trial design (e.g., parallel, cluster, noninferiority)	If the unit of random assignment is not the individual, refer to CONSORT for Cluster Randomized Trials and report the items included in its extension for abstracts	3
Methods			
Participants	Eligibility criteria for participants and the settings where the data were collected	When applicable, the eligibility criteria for the setting of the intervention delivery and the eligibility criteria for the persons who delivered the interventions	3
Interventions	Interventions intended for each group		3
Objective	Specific objective or hypothesis	If pre-specified, how the intervention was hypothesized to work	3
Outcomes	Clearly defined primary outcome for this report		3
Randomization	How participants were allocated to interventions		3
Awareness of assignment	Who was aware of intervention assignment after allocation (for example, participants, providers, those assessing outcomes), and how any masking was done		3
Results			
Number randomly assigned	Number randomized to each group		3
Recruitment	Trial status		3–4
Interventions		Extent to which interventions were actually delivered by providers and taken up by participants as planned	3–4
Number analyzed	Number analyzed in each group		N/A
Outcomes	For the primary outcome, a result for each group and the estimated effect size and its precision		N/A
Harms	Important adverse events or side effects		N/A
Conclusions	General interpretation of the results		N/A
Trial registration	Registration number and name of trial register		4
Funding	Source of funding		4

**Table 3:** CONSORT 2010 items and CONSORT-SPI 2018 checklist for the main text

**Section**	**Item #**	**CONSORT-SPI 2010**	**CONSORT-SPI 2018**	**Reported on page #**
**Title and abstract**				
	1a	Identification as a randomized trial in the title^§^		1
	1b	Structured summary of trial design, methods, results, and conclusions (for specific guidance see CONSORT for Abstracts)^§^	Refer to CONSORT extension for social and psychological intervention trial abstracts	2
**Introduction**				
Background and objectives	2a	Scientific background and explanation of rationale ^§^		6-8
	2b	Specific objectives or hypotheses ^§^	If pre-specified, how the intervention was hypothesized to work	6-8
**Methods**				
Trial design	3a	Describe of trial design (such as parallel, factorial), including allocation ratio ^§^	If the unit of random assignment is not the individual, please refer to CONSORT for Cluster Randomized Trials	9
	3b	Important changes to methods after trial commencement (such as eligibility criteria), with reasons		N/A
Participants	4a	Eligibility criteria for participants^§^	When applicable, eligibility criteria for settings and those delivering the interventions	10
	4b	Settings and locations where the data were collected		10
Interventions	5	The interventions for each group with sufficient details to allow replication, including how and when they are actually administered ^§^		16-17
	5a		Extent to which interventions were actually delivered by providers and taken up by participants as planned	N/A
	5b		Where other informational materials about delivering the intervention can be accessed	
	5c		When applicable, how intervention providers were assigned to each group	
Outcomes	6a	Completely defined pre-specified outcomes, including how and when they were assessed^§^		12-15
	6b	Any changes to trial outcomes after the trial commenced, with reasons		N/A
Sample size	7a	How sample size was determined^§^		11-12
	7b	When applicable, explanation of any interim analyses and stopping guidelines		N/A
**Randomization**				
Sequence generation	8a	Method used to generate the random allocation sequence		11-12
	8b	Type of randomization; detail of any restriction (such as blocking and block size)^§^		11-12
Allocation concealment mechanism	9	Mechanism used to implement the random allocation sequence, describing any steps taken to conceal the sequence until interventions were assigned^§^		11-12
Implementation	10	Who generated the random allocation sequence, who enrolled participants, and who assigned participants to interventions^§^		DRL generated the random allocation. AMK, CL, AGS, AM, NZ, MS, KS, YJG, BAK, MJO, ES, ADW, and JEC enrolled and assigned participants.
Awareness of assignment	11a	Who was aware of intervention assignment after allocation (for example, participants, providers, those assessing outcomes), and how any masking was done		DRL, AGS, AM, NZ, MS, and KS.
	11b	If relevant, description of the similarity of interventions		N/A
Analytical methods	12a	Statistical methods used to compare group outcomes^§^	How missing data were handled, with details of any imputation method	20-21
	12b	Methods for additional analyses, such as subgroup analyses, adjusted analyses, and process evaluations		20-21
**Results**				
Participant flow (a diagram is strongly recommended)	13a	For each group, the numbers randomly assigned, receiving the intended intervention, and analyzed for the outcomes^§^	Where possible, the number approached, screened, and eligible prior to random assignment, with reasons for non-enrolment	Fig 1
	13b	For each group, losses and exclusions after randomization, together with reasons^§^		22
Recruitment	14a	Dates defining the periods of recruitment and follow-up		N/A
	14b	Why the trial ended or was stopped		N/A
Baseline data	15	A table showing baseline characteristics for each group^§^	Include socioeconomic variables where applicable	N/A
Numbers analyzed	16	For each group, number included in each analysis and whether the analysis was by original assigned groups^§^		N/A
Outcomes and estimation	17a	For each outcome, results for each group, and the estimated effect size and its precision (such as 95% confidence interval)^§^	Indicate availability of trial data	N/A
	17b	For binary outcomes, the presentation of both absolute and relative effect sizes is recommended		N/A
Ancillary analyses	18	Results of any other analyses performed, including subgroup analyses, adjusted analyses, and process evaluations, distinguishing pre-specified from exploratory		N/A
Harms	19	All important harms or unintended effects in each group (for specific guidance see CONSORT for Harms)		N/A
**Discussion**				
Limitations	20	Summarize the main results (including an overview of concepts, themes, and types of evidence available), link to the review questions and objectives, and consider the relevance to key groups.	Trial limitations, addressing sources of potential bias, imprecision, and, if relevant, multiplicity of analyses	N/A
Generalizability	21	Discuss the limitations of the scoping review process.	Generalizability (external validity, applicability) of the trial findings^§^	N/A
Interpretation	22	Provide a general interpretation of the results with respect to the review questions and objectives, as well as potential implications and/or next steps.	Interpretation consistent with results, balancing benefits and harms, and considering other relevant evidence	N/A
**Important information**				
Registration	23	Registration number and name of trial registry		21
Protocol	24	Where the full trial protocol can be accessed, if available		21
Declaration of Interests	25	Sources of funding and other support; role of funders	Declaration of any other potential interests	21
Stakeholder investments	26a		Any involvement of the intervention developer in the design, conduct, analysis, or reporting of the trial	N/A
	26b		Other stakeholder involvement in trial design, conduct, or analyses	N/A
	26c		Incentives offered as part of the trial	N/A

All patients will be assessed either in person at the initial visit or via telemedicine technologies (text or video) at baseline, on the day of surgery, and 1, 2, and 3 months after the surgery. We will evaluate how patients comply with and respond to RxWell together with weekly supports. We will then determine the program's efficacy in ameliorating symptoms.

### Study participants

#### Sampling method

The target population is patients undergoing elective primary total hip or primary total knee replacements at UPMC (Shadyside, Magee, Passavant, and East) and are found to have high levels of anxiety and/or depression using validated surveys. We will use the Patient-Reported Outcomes Measurement Information System (PROMIS) scales for the assessment of anxiety and depression. The sampling method will be a convenience sampling.

##### Inclusion criteria

Eligible participants will be adult patients undergoing primary total hip or knee arthroplasty for a degenerative condition, who can read and speak English, with access to a smart phone or tablet and with a T-score ≥ 60 in short PROMIS Anxiety and/or Depression short forms 4a.

##### Exclusion criteria

Patients will be excluded from the study if they meet any of the following criteria: plans to undergo a non-elective surgery or secondary arthroplasty; profound mood disorder that requires emergent care, defined as a *T*-score > 70 in PROMIS Anxiety 4a and/or Depression 4a forms, neurocognitive impairment, dementia or active delirium, or severe intellectual disability; and no access to a smartphone or tablet.

#### Sample size calculation

This is a pilot study meant to inform a subsequent RCT based on this treatment. We will determine if the study is a success if this effect size is observed in our primary outcomes and thus our study is powered based on these effects. From observed estimates in concurrent studies, we expect a clinically significant effect size of Cohen’s D greater than or equal to 0.8. Our primary analysis is comparing PROMIS T scores, GAD-7 and PHQ-8 between groups on day of surgery. Therefore, with 95% confidence, 80% power, using two sided tests and assuming a 50% retention rate, our desired sample size is 136. We will utilize 1:3 allocation, randomizing 34 to the control group and 102 to the intervention group. We are allocating 1:3 to get more accurate estimates around within treatment group improvements as well as to increase the number of patients we can potentially aid by supplying them with the low-risk treatment. The 1:3 allocation was chosen based on the study design and the nature of the intervention. This is a proof-of-concept study in which we want to evaluate the feasibility of RxWell™. In addition, our target subjects are patients with a moderate level of anxiety and/or depression, and we consider it is more ethical to offer a treatment to as many subjects as possible. Sample size calculations were conducted using the Power Analysis and Sample Size Software (PASS - 2022). NCSS, LLC. Kaysville, Utah, USA.

### Outcome measures

We will assess the following:

- RxWell™’s acceptance by practitioners and patients will be measured by qualitative interviews among all practitioners and randomly selected patients.
- Workflow changes brought on by the implementation of RxWell™, and
- Impact of RxWell™, on mood disorders (anxiety, depression, pain catastrophizing) in the immediate pre- and post-operative periods.

We will also test the impact of RxWell™, on

- Sleep disorders
- Fatigue
- Ability to participate in social roles
- Pain interference
- Cognitive function
- Perioperative pain
- Opioid requirements and opioid morphine equivalents,
- Functional recovery, and
- Resource utilization associated with the surgery and recovery (hospital length of stay, duration of physical therapy).

Ultimately, our outcome is to determine if RxWell™ can improve surgical outcomes.

Primary and secondary outcomes will be measured at baseline preoperatively, on the day of the surgery, and 1, 2, and 3 months after surgery (Table 1). Preoperative data will be collected virtually via REDCap surveys by study team members. Postoperative data collection will be performed via email surveys and telephone calls by a member of the study team. Research coordinators collecting outcomes will not be blinded to group assignment. Patients will not be blinded and will be informed that the study aims to assess the effect of dCBI in addition to usual care compared to usual care alone on surgical patients.

#### Primary outcomes

The primary outcome is the patient’s score on the PROMIS Anxiety 4a Questionnaire and on the PROMIS Depression 4a Questionnaire, as well as on the GAD-7 and PHQ-8 scales 1 month before surgery, on the day of the surgery, and 1, 2, and 3 months after surgery, to assess the severity of the patient anxiety and depression levels and the potential effect of RxWell™ on these conditions (Table 1).

The short PROMIS 4a questionnaires are 4-item forms with five-response options per question ranging in value from one to five. The lowest possible raw score is 4, and the highest possible raw score is 20. In both cases, a higher score indicates worse status, and both assess the condition over the past 7 days. The PROMIS Anxiety instruments measure self-reported fear (fearfulness, panic), anxious misery (worry, dread), hyperarousal (tension, nervousness, restlessness), and somatic symptoms related to arousal (racing heart, dizziness). The PROMIS Depression instrument measures self-reported negative mood (sadness, guilt), views of self (self-criticism, worthlessness), social cognition (loneliness, interpersonal alienation), and decreased positive effect and engagement (loss of interest, meaning and purpose). The PROMIS scores are centered with a mean of 50 and standard deviation of 10. The *T*-score range for severe anxiety is > 70, moderate 60–69, mild 55–59, and < 55 is none to less anxiety. Based on our prior work, we chose a baseline anxiety and depression mean *T*-score of 60, with a standard deviation of 10.

The Generalized Anxiety Disorder (GAD-7) is a 7-question instrument that measures self-reported anxiety, worry, trouble relaxing, restlessness, irritability, and fear in the last 2 weeks [32]. The Patient Health Questionnaire (PHQ-8) is an 8-question instrument that measures self-reported interest and pleasure, irritability and depressed mood, problems with sleep, appetite and concentration, lack of energy, changes in the speed of movement and speech, and low self-esteem, over the last 2 weeks [33]. In both questionnaires, there are options for each question, ranging from 0 to 3, and in all cases, a higher score indicates a worse status. The lowest possible score is 0 and the highest total score is 21 for GAD-7 and 24 for PHQ-8.

#### Secondary outcomes

- Pain catastrophizing
- Sleep disorders
- Fatigue
- Ability to participate in social roles
- Pain interference
- Perioperative pain at rest and during movement
- Opioid requirements and opioid morphine equivalents,
- Functional recovery, and
- Resource utilization associated with surgery and recovery (hospital length of stay, duration of physical therapy). We will adjust for surgical decision during the resource utilization modeling as surgeons vary in their decision algorithms for patient discharge timing.

In addition, as part of secondary outcomes, we will record participants’ usage of RxWell, adherence, use frequency, number of techniques completed, and the path in the RxWell platform (anxiety vs. depression) the patient is utilizing. Participants will also complete a satisfaction survey at the end of their participation. The instruments and specific timepoints at which these outcomes are measured are collected in Table 1. The study team will receive monthly reports providing the number of techniques completed, the number of messages to the coach, and the program used in *RxWell* (anxiety vs. depression).

The tools used are as follows:

- Pain catastrophizing—Pain Catastrophizing Scale [34]
- Sleep disorders—PROMIS 29+2 [35]
- Fatigue—PROMIS 29+2 [35]
- Ability to participate in social roles—PROMIS 29+2 [35]
- Pain Interference—PROMIS 29+2 [35]
- Perioperative pain at rest and during movement—pain medications [36]

#### Plans for assessment and collection of outcomes

The study coordinators undergo a week of orientation towards patient screening, approach to patients, and applying the questionnaires. The study coordinators evaluate the data for any quality issues such as duplicate measurements. The questionnaires are validated in prior work [32, 33, 37].

#### Other variables of interest

At baseline, participants will also complete a demographic, lifestyle, and diagnostic survey, consisting of age, gender, ethnicity, race, height, weight, smoking and alcohol use, medical history, ongoing treatments, and comorbidities.

The following medical data will be gathered: type of operation, surgical indication, and American Society of Anesthesiologists (ASA) classification and if the patient received a nerve block before the surgery.

### Study interventions

#### SuRxgWell Trial (the use of RxWell™ among orthopedic surgery patients—digital Cognitive Behavioral Intervention)

The dCBI is a mobile application that guides patients through a series of cognitive behavioral intervention learnings and techniques such as relaxation, cognitive reframing, exposure, and mindfulness. The RxWell™ digital behavioral tool offers a patient access to a live coach via an asynchronous text messaging component within the application. This personalized experience helps guide and motivate the patient through the program and to apply the techniques into everyday life situations. The application provides feedback and progress to the patient. Feedback includes tracking depression and anxiety from Generalized Anxiety Disorder Scale (GAD) and Patient Health Questionnaire (PHQ) measures that are completed within the app. Coaches review messages and scores within 2 business days. In the event of a risk escalation, coaches will email the coach supervisor and include “urgent” in the subject line to notify the supervisor of a high-risk situation or of an interaction with a user where they encounter concerning symptoms. Concerning symptoms may be exhibited either within the context of a message sent to the coach or free text responses within techniques.

This application guides patients through a series of CBT learnings and techniques such as relaxation, cognitive reframing, problem solving skills, distress tolerance, and mindfulness. These techniques are brief and interactive with easy-to-use material presented as audio, video, and interactive text content. The application includes two different pathways, one focuses on anxiety and one on depression management. The participant will utilize one of the programs depending on the initial psychological assessment. If participants score ≥ 60 on the PROMIS Anxiety questionnaire, they will follow the Anxiety Pathway, and if participants score ≥ 60 on the PROMIS Depression questionnaire, they will follow the Depression Pathway. Participants who score ≥ 60 on both questionnaires will be indicated to follow the Anxiety Pathway, and the digital behavioral health coaches will personalize their care by using some depression management techniques at their discretion.

Participants will have access to the live coach via an asynchronous text messaging component within the application, receiving patient-centered and personalized support. The main goal of the coach is to help guide the participant through the application to apply the techniques into everyday life situations and especially into the perioperative context. Coaches can also personalize the program the participant receives and add techniques from the other program if needed.

Although participants can text the coach anytime, the application is not designed to be a crisis management tool. In case of emergency, participants can use a button available within the app which will direct them to contact *ReSolve* or 911. ReSolve is a crisis-management hotline and walk-in clinic as part of the UPMC.

#### Usual care

The control intervention consists of the usual care of a patient undergoing primary total hip or knee replacement, which includes a surgery-specific education session before surgery. This pre-surgical visit provides a detailed verbal information on the preparation for surgery in terms of nutrition, expectations, breathing exercises, and usual workflow until the day of surgery; surgery itself; and materials used for the replacement and recovery from surgery. Questions and concerns raised by patients are also answered and discussed by the orthopedic surgery nurse coordinator. Patients will also be educated about the potential resources for anxiety and depression management.

### Data collection procedure (recruitment and data collection procedure)

#### Recruitment and retention

Patients scheduled for their pre-operative evaluation at the Center for Perioperative Care clinics or at the orthopedic clinic will be screened for eligibility, and those deemed eligible will be approached by the study coordinators for recruitment or given a flyer by nurse practitioners or physician assistants. Patients who are interested will be able to consent with an investigator or a research coordinator in person, reach out to the research team, and use the QR code that appears on the flyer to give their contact information, or they will be called by a study team member.

Participants will be able to sign the informed consent via REDCap through a virtual interview with the principal investigator or in person with an investigator or research coordinator. In the case of the e-consent, virtual interviews will be conducted remotely using HIPAA-compliant *Zoom* and following a semi-structured format to ensure a systematic yet flexible approach. On the consent form, participants will be asked if they agree to use of their data should they choose to withdraw from the trial. Participants will also be asked for permission for the research team to share relevant data with people from the university taking part in the research or from regulatory authorities, where relevant. This trial does not involve collecting biological specimens for storage.

Once patients provide consent, we will use the PROMIS questionnaires to assess their mood condition and identify those with high levels of anxiety and depression. They will be eligible if they have a *T*-score ≥ 60 on the PROMIS Anxiety Short Form 4a v1.0 questionnaire and/or PROMIS Depression 4a Short Form v1.0 questionnaire. Participants who meet this criterion will be enrolled and randomized into perioperative treatment with a RxWell™ and usual care or only usual care.

To promote participant retention and compete follow-up, the weekly trial meetings will identify potential data loss and prompt the study coordinators and coaches to reach out to subjects to engage them.

#### Surveys employed

The full survey, including the four questionnaires, is expected to be completed in no more than 15 min. REDCap will host all surveys.

#### Data collection

Initially, participants will complete a demographic, lifestyle, and diagnostic survey, consisting of age, gender, ethnicity, race, height, weight, smoking/alcohol use, medical history, ongoing treatments, and comorbidities.

At the baseline, participants from both groups (intervention and education group) will also complete the PROMIS 29+2 v2.0 form, except the Anxiety and Depression subsection since it will have been already completed during the eligibility assessment, the GAD-7 and PHQ-8 scales, the Pain Catastrophizing Scale (PCS), the Knee injury and Osteoarthritis Outcome Score (KOOS), and Hip injury and Osteoarthritis Outcome Score (HOOS) and the Pain Medications Survey and Pain Scores Survey created in REDCap (Table 1).

Participants will be assessed with the same instruments on the day of surgery. In addition, participants allocated in the intervention group will be assessed with the GAD-7 and PHQ-8 bi-weekly through the RxWell™ application during its use.

#### Long-term follow-up

Both groups will be assessed at 1, 2, and 3 months after the surgery, which corresponds to 2, 3, and 4 months after the enrollment. Participants from both groups will have to complete the same instruments described above and the post-surgical survey created in REDCap, which records potential post-surgical complications and additional measures like the length of stay in the hospital, the time to ambulation, and the use of physiotherapy. In addition, participants will also have to complete a satisfaction survey about RxWell™ at the 3-month timepoint, corresponding to the last interaction with the participants. The purpose of this follow-up is to collect data on long-term effects of the interventions and document any changes that might take place in the participant’s disease condition, their frequency of CBI intervention practice, and other additional changes noted.

Users will complete GAD-7and PHQ-8 bi-weekly to monitor their behavioral health over time, and the digital behavioral health coaches will review the scores, free text in each technique, and user message to the coach within two business days. In the event of a risk escalation, coaches will contact the coach supervisor to notify a high-risk situation or an interaction with a user where they encounter concerning symptoms that may be exhibited either within the context of a message sent to the coach or free text within a technique.

As for behavioral studies, trials are commonly designed with a waitlist control group. This approach would allow that all participants have an opportunity to utilize the intervention; however, in the current trial with a focus on feasibility, we will use the randomized approach and plan the waitlisted approach for a larger clinical trial.

The data entry and coding will be performed by study coordinators electronically using the REDCap. All the data are secured behind a firewall within the University of Pittsburgh (Clinical and Translational Science Institute at the University of Pittsburgh Grant Number UL1-TR-001857).

### Data analysis

The primary hypotheses and all other comparisons will be assessed with intention to treat estimates. Between-group differences in primary outcomes, PROMIS anxiety and depression scores, PHQ-8, and GAD-7 scores will be tested using independent two sample *t*-tests on the day of the surgery. Two-sided tests using *p* values of < 0.05 will be considered statistically significant. Differences of tests showing Cohen’s *D* of > = 0.8 will be considered clinically significant. Participants with missing primary timepoint data will be excluded from testing. For secondary outcomes, we will calculate descriptive statistics i.e., usage of RxWell™, adherence, use frequency, etc. Testing of treatment and control group differences in secondary outcomes will be applied to appropriate variables, i.e., participant perioperative pain, opioid equivalent requirement, functional recovery, etc., for time point baseline, day of surgery, and 1 month follow-up. Continuous variables will be described using means and standard deviations. Categorical variables will be described using frequencies/proportions. We will use independent sample *t*-tests and chi-squared analyses to examine between group differences. Fisher’s exact tests and Mann-Whitney *U* tests will be performed in replacement of their counterpart parametric tests where appropriate. Missing data will be removed from denominators, proportions, distributions, and testing of their differences. We will use longitudinal mixed effect models to account for within person variance across all time points as a secondary analysis.

Baseline patient demographics, comorbidities, and procedure details will be stratified by group, and described standardized mean differences will be calculated to compare groups (Table 1). As a secondary analysis, we will use longitudinal mixed effect models to account for within person variance across all time points. We will adjust models using demographics found to have greater than 0.2 standardized mean difference. No subgroup analysis will take place.

We estimated that 50% or more of eligible patients will be enrolled, and as for the proportion of engagement, we are targeting 70% or more of the enrolled patients attending the first SuRxgWell™ orientation class and 50% or more of the follow-up weekly. As for the proportion of compliance, we are targeting 60% or more of the enrolled participants completing the questionnaires; as for the proportion of adherence, we are targeting 60% or more of the enrolled participants who are considered engaged with the app, completing three or more CBT techniques.

### Ethical considerations

#### Criteria for discontinuing or modifying allocated interventions

Our intervention is low risk/low harm, and we will discontinue allocated intervention if the subjects change their decision to be part of the trial.

#### Compliance and adherence

Bi-weekly surveys will obtain compliance and adherence data on the use of RxWell™ platform.

## Coordinating center and trial steering committee

### Coordinating center

The study coordinating center is headed by the director of clinical research operations overseeing two regulatory specialist and three study coordinators assigned for this trial. The principal investigator is part of the coordinating center as well.

The director of clinical research operations has more than 20 years of successful experience in clinical trials and is responsible for successful execution of the trial, answer day-to-day questions by the personnel, and communicate with the surgical offices.

The regulatory specialists prepare and update the institutional review board, clinical trials federal website, and university communications. They are responsible for timely communications with the overseeing bodies and compliance.

The study coordinators screen potential subjects, approach them in the clinic, and perform the surveys in person or over telemedicine platforms. The study coordinators are also responsible for timely and ethically entering clinical trial data.

The principal investigator (AMK) is available for all day-to-day questions via electronic communications from any and all of the members of the coordinating center.

### Trial steering committee

The trail steering committee is headed by the principal investigator. The other member of the steering committee are as follows:

- Director of clinical research operations,
- Regulatory specialists,
- Study coordinators,
- Representatives from the RxWell research group and coaches,
- Other investigators,
- Statistical consultant, and
- Orthopedic surgical consultants.

The principal investigator is responsible for the ethical, efficient, and sustainable execution of the trial. The principal investigator also ensures a healthy communication between various stakeholders.

The RxWell researchers and coaches have a long-standing experience in coordinating research projects for cognitive behavioral interventions. They ensure that the electronic application (“app”) is downloaded and used by the subjects and guide them if needed through all the steps. They also produce weekly compliance and use data.

The other senior investigators have full commitment based on their clinical and research experience for this trial and provide valuable insight. They also provide direct feedback to the principal investigator.

The statistical consultant was instrumental during the creation of the protocol and is available for other input during the trial.

The orthopedic surgical consultants provide clinical feedback about this trial based on their patient interactions in the perioperative period.

### Sponsor involvement

The sponsor is Prof. Dr. Aman Mahajan, chair, Department of Anesthesiology and Perioperative Medicine. Prof. Mahajan ensured that proper arrangements are in place to initiate, manage, and report for the SuRxgWell trial. The sponsor played no part in study design; collection, management, analysis, and interpretation of data; writing of the report; and the decision to submit the report for publication.

### Dissemination

The results of this study will be communicated to the participating clinics and published in peer-reviewed publications and presented at national, clinical, and scientific conferences or meetings.

## Discussion

Up to 43% of post-TKA patients report persistent pain, functional limitations, and poor quality of life despite clinical and radiological indicators of surgical success [38, 39]. Greater symptom intensity and movement intolerance after surgery is usually attributed to surgical technique or implant design [40, 41]. However, the evidence suggests these aspects have minimal influence and that inadequately addressed mental and social health distresses could be important factors [42]. Giesinger et al. documented that psychological and demographic factors accounted for more variance in patient-reported outcomes than surgical factors after hip and knee arthroplasty [43]. Depressed patients remain up to a 6-times-higher risk for dissatisfaction after primary TKA than patients who are not depressed independent of surgical recovery, and anxiety has demonstrated to increase pain and postsurgical complications [44–46].

The prevalence of clinically meaningful anxiety or depression symptoms is 6–7% in the USA, but in patients undergoing TKA, these values increase up to 20% [47, 48]. The current practice in elective orthopedics does not involve routine psychological interventions, but given the high prevalence of psychological distresses in patients undergoing arthroplasty and the impact of these factors in its outcomes and patient perception, there is a need for an increased understanding and perioperative assessment of the psychological condition in these patients [49, 50].

Cognitive behavioral intervention is an effective psychological treatment for depression and anxiety. It carries the potential of reducing depression, anxiety, pain catastrophizing, and postsurgical pain in patients undergoing total arthroplasty [27, 51, 52].

Similar to our proposal, das Nair determined the feasibility of conducting a trial of home-based, in-person, pre-surgical psychological intervention based on CBT and its effect in terms of mood, pain, and function in patients undergoing TKA, and at 6-month follow-up, the patients allocated to the intervention group showed a significant benefit in mood, pain and functionality [53].

There is preliminary work assessing the feasibility of telemedicine in perioperative care of surgical patients, but the evidence for orthopedic surgery, and especially incorporating a psychological intervention, is limited [54–56]. Buvadendran and his colleagues studied the effect of CBI in patients undergoing TKA and proved that its use prior to surgery, both in person or via telehealth, lead to reduced pain catastrophizing scores in postsurgical stages, supporting the results found in previous literature [23, 25, 26, 57, 58]. Rognsvåg et al. developed an Internet-delivered cognitive behavioral therapy program for use in combination with exercise and education in patients at increased risk of chronic pain following total knee arthroplasty [59]. Antony et al. also conducted a randomized clinical trial to evaluate of the effectiveness of acceptance and commitment therapy delivered via a mobile phone messaging robot to patients who had their THA or TKA postponed due to COVID-19 pandemic, showing better surgical outcomes in participants from the intervention group [60].

To the best of our knowledge, our current proposal is the first randomized controlled trial using dCBI before, during, and after surgery in patients undergoing both THR and TKR. Studies demonstrated the feasibility of using telemedicine to provide orthopedic consultations to patients living in remote areas safely without serious adverse events increase patient satisfaction, reduce travel, time, and costs [61–69]. Interestingly, during COVID-19 pandemic, patients with anxiety or depression were more likely to have a telehealth encounter than an in-person encounter [70]. We also foresee limitations in this study, such as limited access to the required technology and those living in rural or underserved areas are less likely to use telehealth, emphasizing the need for attention to at-risk populations in future trials [55, 71–73]. As we incorporate dCBI into our practice, we envision embedding dCBI into surgical care models.

### Supplementary Information

**Additional file 1.**

## Data Availability

The results of the trial data will be presented in the main manuscript(s) as additional supporting files in a machine-readable format.
